# Barriers to Skin Cancer Diagnosis and Treatment in Low‐ and Middle‐Income Countries and Solutions: A Literature Review

**DOI:** 10.1002/puh2.70042

**Published:** 2025-03-11

**Authors:** Christopher Maatouk, Don Eliseo Lucero‐Prisno

**Affiliations:** ^1^ Faculty of Medical Sciences Lebanese University Hadath Lebanon; ^2^ Department of Global Health and Development Faculty of Public Health and Policy London School of Hygiene and Tropical Medicine London UK; ^3^ Office for Research Innovation and Extension Services Southern Leyte State University Sogod Southern Leyte Philippines; ^4^ Research and Innovation Office Biliran Province State University Naval Philippines

**Keywords:** barriers, diagnosis, low‐income, middle‐income, skin cancer, solutions, treatment

## Abstract

Skin cancer mortality disproportionately affects low‐ and lower‐middle‐income regions despite the prevalence being lower than in high‐income countries. Considering the need to diagnose it early for the best outcomes, this review addresses the barriers preventing it from being diagnosed and treated promptly and proposes possible solutions.

Some of the barriers we found include the low number of dermatologists and pathologists, inadequate facilities, lack of education, the cost of healthcare, the denial of needing professional help, the fear and stigmatization of a skin cancer diagnosis, and the reliance on non‐medical therapies. Meanwhile, solutions we identified are training programs for healthcare professionals and the public, technological advancements (including nanotechnology‐based treatments, telemedicine, and social media use, the development and implementation of artificial intelligence programs), international collaborations, research, and increasing the number of cancer registries and national cancer control plans.

Despite these solutions not being foolproof, they will lead to earlier cancer diagnosis, more individuals seeking skin check‐ups, better knowledge of skin cancer, improving the quality of life of vulnerable populations, and decrease in mortality.

## 1 Introduction

Public health is a multidisciplinary field concerning all levels of the public, from researchers to policymakers. The aim is to promote health at the population level rather than just the individual. It encompasses disease prevention, life quality, and life prolongation through the efforts of organizations and various public and private communities [1]. This goal is usually achieved through education and health policy changes. Global health is an extension of this concept but on a broader international level. It calls for changes to happen from a medical physician level (through disease prevention, e.g.) up to changes in government policies to positively influence the health of millions of people [2].

Global health brings into the limelight the disparities among different countries, particularly different income levels. The World Bank defines low‐income countries as those with a gross national income per capita of $1135 or less in 2022, whereas lower‐middle‐income countries were between $1136 and $4465 [3]. Due to widespread poverty, they usually lack adequate healthcare infrastructure. These circumstances create a higher incidence of diseases because treatment is not as available as in higher income countries. Chronic diseases also follow the same trajectory as they become more prevalent due to limited resources [4]. This environment has led certain low‐ and middle‐income countries (LMICs), for example, to have a prevalence of skin disease between 50% and 80% [5].

Among these chronic diseases is skin cancer, which is usually divided into melanoma and non‐melanoma (including basal cell carcinoma and squamous cell carcinoma). Melanoma is less prevalent but more fatal, whereas non‐melanoma skin cancers (NMSCs) are more prevalent yet less fatal [6]. Cancer registries do not usually track non‐melanoma cancers because they may go undiagnosed or are usually successfully treated by surgery or ablation. Therefore, the incidence of skin cancer is often underestimated [7]. According to the WHO, there were an estimated 1.5 million new cases of skin cancer in 2020. Of these new cases, 325,000 were attributed to melanoma, which caused around 57,000 deaths that same year [8]. The first step of diagnosis is usually visual inspection following the ABCDE rule (asymmetry, border irregularity, color variation, diameter, and evolving size, shape, or color). This approach might be followed up by dermoscopy or biopsy (the gold standard for diagnosis) [9]. Once confirmed, treatment depends on the cancer's characteristics (e.g., type, size, location, staging). Treatment ranges from topical chemotherapeutic creams and surgical excision to additional chemotherapy or radiotherapy [10], as well as new emerging treatments utilizing nanotechnology, which we will expand on further in this review.

Moreover, the disparities in skin cancer outcomes between low‐income and higher income countries are stark. This reality is due to a myriad of reasons, including a lack of awareness, insufficient training, inadequate access to diagnostic material and treatment facilities, and the lack of comprehensive cancer registries [11]. This review aims to explain the barriers to diagnosis and treatment of skin cancers in low‐income countries. It will also investigate potential solutions to reduce the burden of skin cancer in these poorer regions.

## 2 Effect of Skin Cancer

On the one hand, cancer has been linked to poverty in previous studies. Even in the United States, low‐income counties showed higher cancer mortality. Some of the reasons mentioned were food insecurity, alcohol, nutrition, low‐quality care, smoking, physical inactivity, obesity [12, 13], and lower education [14]. On the other hand, skin diseases are proven to take a toll on affected individuals’ quality of life [15, 16, 17] and mental health [17, 18]. Possible treatment side effects also bolster depression and anxiety. Therefore, when combined together, skin cancers can seriously affect the lives of those who are afflicted. In the case of melanoma, despite having lower numbers, low‐income countries have disproportionate numbers of deaths [19, 20]. In fact, over 70% of deaths caused by cancer take place in LMICs [21]. By 2040, an estimated 11.5 million new cancer diagnoses will arise in LMICs (+158% in lower‐middle‐income countries and +99% in low‐income countries, whereas it is only +38% in high‐middle‐income countries) [22].

In order to understand how low‐income populations are affected by skin cancers, it is essential to present the risk factors for each group of cancer. Risk factors for melanoma include UV radiation [23, 24], genetic susceptibility [24, 25, 26, 27], fair skin, light hair, green or blue eyes [28, 29], aging [30], personal medical history, and family history [31, 32, 33]. Transplant recipients, especially if immunocompromised, have over twice the risk of developing melanoma compared to the rest of the population [34]. Additionally, the higher number of freckles and raised nevi are risk factors [35]. Women are affected more in the younger population than men, but the reverse is true in the older population [32]. One of the reasons for the higher number of melanoma cases in higher income countries may be related to sunbathing or the increased use of sunbeds for tanning, with a 75% increased risk of melanoma in those under 35 with a history of sunbed use [36]. Furthermore, poor tanners are more susceptible to sunburns. Individuals who, when attempting to tan, receive severe sunburns have an almost six‐fold risk of developing melanoma.

The risk factors for non‐melanoma cancers include similar factors to the melanoma risk factors (UV light [23, 24, 37], age [37, 38], fair skin, immunosuppression [39], organ transplant [37, 40–42], and personal and family medical history). They also include being male (relating to the increased sun exposure) [37], certain chemicals (e.g., arsenic, coal tar, and paraffin), radiation exposure, and genetic syndromes (e.g., Fanconi anemia, Bloom syndrome), among others [43, 44, 45].

When analyzing how skin cancer affects low‐income countries, we note that living in poor neighborhoods was associated with a 2%–4% disadvantage in melanoma survival [46]. A particular study points out the different trajectories to compare the differences between high‐ and low‐income regions: Between 1990 and 2019, NMSC had increased in cases, deaths, and disability‐adjusted life years (DALYs) worldwide. However, high‐middle SDI regions saw a decrease in the age‐standardized mortality rate, whereas high‐SDI regions saw it remain unchanged. Overall, the higher prevalence in higher income countries is possibly due to an increased susceptibility to developing melanoma, whereas the higher mortality in lower‐income areas may be explained by the lack of access to educational campaigns, screenings, and treatment [47].

## 3 Barriers to Diagnosis and Treatment

The most glaring barrier is the evident paucity of dermatologists in certain low‐income countries. For example, of the 55 African countries, 30 have no dermatology training programs. In fact, in most of the continent, there is less than 1 dermatologist per million population, whereas the United Kingdom has 10, the United States has 36, and Germany has 65 dermatologists per million [48]. The lack of access to such healthcare professionals in low‐income countries has reinforced the prevalence of skin diseases [15]. This deficit naturally leads to fewer chances of physical examinations, decreasing the chance of being diagnosed and treated. Another missing healthcare professional in this equation is the pathologist, with some areas, such as the sub‐Saharan region, having less than one per 500,000, compared to the United States, which has one pathologist for every 15,000–20,000 people [49]. These circumstances create large areas of medically underserved communities, typically in rural areas, while leaving few and overworked professionals in urban areas. Furthermore, LMICs lack oncologists, radiotherapists, and physicians with geriatric expertise (because cancer prominently affects older individuals) [50].

The lack of nearby physicians creates a couple of problematic scenarios: Patients will either wait until the symptoms are too severe to ignore until they visit a doctor, or they may rely on other sources of treatment. On the one hand, a study in Nigeria found that participants with a skin rash may choose not to visit a physician due to the distance unless they have a fever. However, should the distance be too great, they would not visit a professional even with a fever [51]. This decision‐making process involves weighing in the observable severity of their symptoms against the cost of traveling long distances, the cost of the check‐up, waiting times, and the cost of treatment. Given the poverty an individual may be facing in this situation, the cost of being treated may outweigh the benefits.

Moreover, this might push the afflicted individual to other resources, such as pharmacists or natural healers. Patients may entrust pharmacists in their skin disease treatments for many reasons, including the inaccessibility of general practitioners, the perceived non‐serious nature of their symptoms, and convenience [52]. Patients may also self‐medicate using medications either bought from the pharmacy or from relatives. In one Tanzanian study discussing self‐medication for dermatological symptoms, the majority of cases actually worsened with time (54%), and around a third (32%) saw no changes in their condition. An estimated 82% of participants in this study were found to have used improper drugs for their cases [53]. At the same time, an Ethiopian study showed that 52% of sick individuals visiting traditional healers used this treatment route as a first‐choice option. Among the reasons they mention are efficacy and dissatisfaction with modern medicine. The majority of traditional healers interviewed claim their knowledge stems from family‐based apprenticeships [54]. Another study on traditional healers and faith‐based healers demonstrated that they do not understand the cause of cancer or the treatment. Although some traditional healers attributed cancer to poor hygiene, eating cassava, and blood impurities, faith‐based healers generally attributed it to demonic or spiritual attacks. Treatment for the first group included drinking herbs or preparations for skin lesions, whereas it consisted of prayers for the second group [55]. All in all, these routes delay the diagnosis and treatment of any emergent cancers, leading to worse prognoses and higher mortality rates.

Additionally, a lack of education and awareness are important factors in the mentioned disparities. A review showed that health literacy was the most frequently mentioned barrier to any type of cancer studied [56]. Should a patient be unaware of the significance of specific symptoms, they might brush it off, hoping they would go away soon enough. The lack of awareness hits low‐income countries precisely due to the decreased resources they may have in terms of schools or higher education. A form of community‐learned medicine replaces modern medicine. Finally, this community‐based life may create a sense of fear of being diagnosed with cancer and being stigmatized by the community for that [57, 58, 59]. Patients may also fear a positive cancer diagnosis, pondering on what lies ahead of such a diagnosis [58, 60]. This fear is also the case for less educated individuals who avoid visiting the doctor in this case because of their fear of having a serious illness (i.e., cancer) and dying [61]. In the case where they believe a treatable type of cancer to be deadly, they may push themselves away from seeking a diagnosis, leading to the progression of the initially treatable disease and worsening the prognosis. This case applies to melanoma, which is highly treatable in the initial stages but becomes dangerous and more fatal as it gets the chance to progress and metastasize. The American Cancer Society states that the 5‐year survival rates for melanoma are as follows: localized (>99%), regional (71%), and distant (32%). These numbers highlight the necessity for early detection and treatment [62].

An article investigating why patients may avoid having their skin symptoms examined noted that the most reported reasons were minimizing health issues (e.g., claiming most people in the area have a disease to deal with), wanting to remain in control (e.g., not wanting others to influence their life decisions), unwillingness to show emotions, privacy concerns, and waiting time at doctors’ offices [63].

These barriers may explain why individuals from lower‐income areas are less likely to consult with their doctors [64]. All in all, they also create an arduous path to treating skin cancers that arise in the population. With the lack of dermatologists and pathologists to diagnose cases of cancer and the barriers preventing patients from presenting to healthcare professionals, the route to improvement appears bleak. Even if patients were willing to go for a check‐up, the lower number of facilities and inadequate facilities for cancer treatment may suppress the chances of successful treatment [65].

## 4 Solutions to the Aforementioned Barriers

### 4.1 Boosting Education and Awareness

Awareness of skin cancer symptoms among the population can be achieved through social media or government‐funded campaigns. However, this endeavor might be challenging in low‐income countries, considering funding for such programs may not be readily available. An article studying the average campaign cost estimated it to be around $156,000 [66]. Other methods of spreading knowledge could be through public booths or flyers with a professional follow‐up intended to reach medically underserved communities. These additional sources of information can help tackle the lack of knowledge and reduce the stigmatization of illness. A study analyzing the use of structured presentations showed that participants with low education significantly benefited. The method consisted of an illustrated chart explaining the process, followed by screeners ensuring that the participants understood the information presented to them. This method was better than simply handing out a leaflet with a brief verbal explanation [67]. The technique also goes a long way in building trust in the medical community by reaching out to these usually underserved populations.

### 4.2 Training Programs

However, targeting the general population is one part of the equation. A better school of thought would be to combine it with targeting healthcare professionals. Public health awareness and training healthcare professionals, though, must be tailored to skin cancer because one approach might prove to be beneficial for one type of cancer while not suitable for another type [68, 69].

Training programs for dermatology specialization should be introduced in greater numbers, which would increase the number of dermatologists available in usually underserved areas. A study also mentions the possibility of connecting pathologists who work alone in desolate areas to tertiary pathology centers where most pathologists are located through telepathology systems that function in real‐time. It also suggests training non‐pathology medical and paramedical staff to perform diagnostic tests, such as fine‐needle aspiration [49]. This additional help would help expedite the diagnostic process and introduce more diagnostic opportunities in PCP clinics.

Training programs for PCPs can also be helpful, especially given the scarcity of dermatologists and pathologists in certain areas. Dermoscopy training may be valuable as it has been shown to decrease unnecessary referrals and increase referrals of actual melanoma cases [70]. This outcome will help reduce the number of patients seen by the dermatologists available and reduce the cost of travel and healthcare on the patient's side. Dermoscopy is also non‐invasive, allowing some patients to forgo unnecessary biopsies, which boosts efficiency and patient comfort and satisfaction. For example, the dermato‐oncological training program (DOTP) showed that trained PCPs provided better lesion descriptions and potential diagnoses in their referral letters and called for fewer unnecessary referrals [71]. A review article mentioned that the best dermatological training programs for PCPs should include an interactive online format allowing busy PCPs to participate and reach a broader number of participants, as well as instructions for dermoscopy and management. It should allow PCPs to help design the training programs. The study also mentions that programs should not be passive and brief [72]. The online nature of the programs would also help in low‐income countries where traveling long distances to assemble PCPs in a particular facility may not be an option.

### 4.3 International Collaborations

International collaborations could be instrumental in this regard, such as the example of the International Society for Geriatric Oncology offering training programs to some LMICs [73] or the African Telederm group. This project virtually connected African partners with the United States and Austria and provided online education for training and research. Focusing the collaboration precisely on the most prevalent diseases in low‐income areas might be constructive. These programs have proven beneficial, such as one program in Mexico against diseases like scabies or the Regional Dermatology Training Center in Africa [74]. Teledermatology can also be a valuable asset, whether or not collaborations with professionals from outside the country are used. One study found melanoma diagnosis was similar between face‐to‐face visits and teledermatology [75]. Telemedicine is also able to correctly identify the majority of malignant lesions as per another article [76]. In another study, a “tele‐triage” system was able to manage a portion of consults by simply using telemedicine. Given adequate photographs, dermatologists inspected and deemed PCP referrals unnecessary 21% of the time for any type of suspected skin cancer and 29% for suspected melanomas [77]. These programs allow for the early detection of malignant lesions and broaden access to dermatologists [78]. By collaborating with healthcare professionals from abroad, local physicians relieve themselves from the overload of cases and stay up to date on the newest treatment options.

Moreover, skin cancer is researched far more often in high‐income regions of the world. Extending this research to low‐income countries could be valuable because risk factors and prevention methods may be different. For example, the advice of avoiding sunbeds in high‐income countries would not be fruitful in low‐income countries because it is not done as often in the latter group. More research in different settings will expand our knowledge of skin cancers and provide a new subset of the world population with more relevant information applicable to them.

### 4.4 Cancer Registries and the Role of Governments

Governments must also do more in the fight against this cancer. The mean per capita health expenditure for high‐income countries was $3224 per person, whereas it was $141 for lower‐middle‐income countries and $39 for low‐income countries. Low‐income and lower‐middle‐income countries have the fewest cancer registries. In fact, only 22% of low‐income countries had a national cancer registry compared to 75% of high‐income countries. Moreover, merely 31% of them had a national cancer control plan compared to 79% of high‐income countries [79]. For example, of the 46 sub‐Saharan countries, only 25 have cancer registries, whereas the cancer estimates of the others must be estimated from neighboring countries. Most of these registries are also located in urban areas, leaving rural areas even more secluded in this health epidemic [74, 75]. The lack of tracking of cancer cases causes a nationwide underestimation of the issue at hand. The information that could be gathered with cancer registries could help describe geographic differences in terms of epidemiology, thereby steering resources needed in treating the most common cancers and forecasting the number of cancer cases that might appear [80, 81]. This solution ties in neatly with the research that should be done in low‐income countries where treatment can also be studied to assess the efficacy in the population. It would also help the governments to lay out detailed and thorough plans to tackle the issues afflicting their people.

### 4.5 Artificial Intelligence (AI) and Modern Technology

AI is slowly encroaching on many professions, though its use in medicine may be slower than in other careers. One might postulate that another solution to the issue at hand may be the development and use of our current modern technology. In fact, some articles have found AI programs to be better than dermatologists in terms of melanoma diagnosis [82, 83, 84]. Despite being unable to replace dermatologists in underserved areas of LMICs completely, it may help accelerate patient physical examination and pathology slide interpretation. An AI program is only as good as the database it is trained upon. Considering that skin cancer primarily affects individuals with fairer skin, it may not be as effective when used on populations of different skin types. Furthermore, programs might be unable to recognize particular skin conditions (e.g., atypical melanoma, NMSC, and precancerous lesions) [85]. Considering that these lesions account for the large majority of skin cancers, the use of AI programs may be limited. Anatomic limitation, including nails, hair‐baring, mucosal, and acral sites, is also a concern [86]. A solution for these issues would be updating the databases regularly to train the program better or limiting the use of AI programs for specific lesions. Moreover, the risk of false positive and false negative results could worsen outcomes. False positives would lead to increased anxiety and increased use of already‐depleted healthcare resources, whereas the latter would prolong the time to receive treatment and worsen health outcomes. Solutions for the risk of false positive results include piloting the AI program in the areas where they will be implemented, and comparing the results to results from biopsies. In contrast, solutions for the risk of false negative results include continuous monitoring by a dermatologist. Overall, any solution will incorporate AI programs to work hand‐in‐hand with dermatologists [86].

Another possible venue for the spread of information is social media. A key issue present on these platforms is the possible misinformation. Healthcare professionals, such as dermatologists in our case, may play a valuable role in the spread of evidence‐based dermatology content. A better informed population about risk factors and suspicious lesion presentation is more likely to seek a professional opinion, helping to detect malignant lesions early and improving health outcomes [87]. In fact, a study noted that exposure to social media content on dermatology skin disease prevention methods prompted many users to seek a face‐to‐face visit, leading to 21.93% of them receiving a new diagnosis, 1.65% of which were a cancer diagnosis [88]. On the other hand, social media can help recruit patients for clinical trials, cutting recruitment costs and timelines. This added benefit would boost information gathering on specific dermatologist‐poor populations such as LMICs and introduce more of the population to the importance of skin checks [89].

Finally, in tandem with the proposed solutions noted above, there needs to be a focus on the continuous development of new and more convenient cancer treatments. New findings like carbonic anhydrase IX (CA IX) allow cancer treatment and mortality reduction [90]. For example, although superficial basal cell carcinoma may be treated non‐invasively with 5‐fluorouracil (5‐FU) or imiquimod with various cure rates, an article mentions curing 31 of 32 lesions with nanoparticles as drug carriers of 5‐FU, prepared by anionic polymerization of butyl‐2‐cyanoacrylate monomer [91]. Despite also being a topical treatment, it showed significantly better cure rates than topical 5‐FU alone of similar duration (96.7% vs. 87.5%) [92].

## 5 Conclusion

In summary, cancer is afflicting most of its burdens on low‐income countries, with 70% of cancer‐related deaths worldwide occurring in these regions. Skin diseases are most rampant in low‐income countries, with some having a 50%–80% prevalence rate. Considering skin cancer ought to be diagnosed early on in the disease, especially in the case of melanoma, due to the fear of metastasis and higher mortality rates, it is imperative to address the barriers to diagnosis and treatment, as well as to propose solutions. Despite skin cancer being less prevalent in low‐income countries, mortality rates are far higher than in their more affluent counterparts. Some of the barriers identified are the evident scarcity of dermatologists and pathologists, the lack of education and awareness of the symptoms of skin cancer, the cost of healthcare and traveling long distances to reach health facilities, the reliance on non‐medical treatments for symptoms (such as self‐medication or natural healers), denial of the need for a medical check‐up, the fear of having a serious illness or death, and the paucity of healthcare facilities precisely in rural areas. Solutions identified to address these barriers include training programs for PCPs (to decrease unnecessary referrals), non‐dermatology staff (such as obtaining biopsies, e.g., fine needle biopsies), and the general population (to raise awareness and education), the use of technological solutions (such as “tele‐triage” and teledermatology, the development and implementation of AI programs, new treatments involving nanotechnology and the use of social media to spread advice from professionals, dispelling misinformation), an international collaboration (for learning and research, which could also expand our knowledge on the risk factors and treatment efficiency in patients besides from the high‐income areas), and expanding the numbers of cancer registries (so that governments can create solid plans and steer funding in the most urgent and prevalent of cases).

## Author Contributions

**Christopher Maatouk**: investigation, writing – original draft, methodology, data curation, writing – review and editing. **Don Eliseo Lucero‐Prisno III**: conceptualization, writing – review and editing, supervision.

## Ethics Statement

An IRB approval was not required as this article is a literature review which did not involve human subjects.

## Conflicts of Interest

The authors declare no conflicts of interest.

## Data Availability

Data sharing is not applicable to this article as no new data were created or analyzed in this study.
